# Case Report: The Association of Wilson Disease in a Patient With Ataxia and GLUT-1 Deficiency

**DOI:** 10.3389/fped.2021.750593

**Published:** 2021-10-05

**Authors:** Jenna Diaz, Ashley G. Fonseca, Richard Arboleda, Alejandro Frade, Maria Pilar Gennaro, Parul Jayakar, Paula Schleifer, Erick Hernandez

**Affiliations:** ^1^Department of Medical Education, Nicklaus Children's Hospital, Miami, FL, United States; ^2^Department of Pediatric Gastroenterology, Hepatology, and Nutrition, Nicklaus Children's Hospital, Miami, FL, United States; ^3^Department of Neurogenetics, Nicklaus Children's Hospital, Miami, FL, United States

**Keywords:** glucose transporter 1 (GLUT1) deficiency, ataxia, elevated liver enzymes, *ATP7B*, *SLC2A1*, case report, Wilson disease

## Abstract

**Background:** Wilson disease (WD) and glucose transporter type 1 (GLUT1) deficiency syndrome are two syndromes with different modes of inheritance but share certain similarities on neurological presentation. To date we have not found previous reports of an association between these two disorders.

**Case Presentation:** Here we describe a 9-year-old male with global developmental delay that presented with intermittent and sudden onset weakness that first occurred at age 3. He was diagnosed with a mutation in the *SLC2A1* (Solute Carrier Family 2 Member 1) gene, which results in GLUT1 deficiency. A ketogenic diet could not be started because of unexplained elevated liver enzymes. Due to his liver enzymes' persistent elevation, further investigations demonstrated mildly decreased ceruloplasmin levels, high basal 24-h urinary copper excretion, and an elevated hepatic parenchymal copper concentration on liver biopsy, consistent with WD. Genetic testing revealed two separate mutations in the *ATP7B* (ATPase Copper Transporting Beta) gene, consistent with WD. The patient was treated with a low copper diet, zinc acetate, and trientine hydrochloride. When liver enzymes normalized, he was subsequently started on a ketogenic diet with improvement in neurological symptoms. His neurological symptoms were most likely secondary to GLUT1 deficiency syndrome, as WD's neurological symptoms are primarily observed in the second decade of life.

**Conclusion:** Recent studies have demonstrated the importance of genetic testing upon unexplained persistent elevation of liver enzymes. This case highlights the importance of carefully evaluating a patient with an unexplained liver disorder, even in the presence of primary neurological disease, as it can have significant therapeutic implications.

## Introduction

Glucose transporter type 1 (GLUT1) deficiency is caused by impaired glucose transport regulation into the brain due to a defect in the *SLC2A1* gene on chromosome 1p34.2 and can be inherited in an autosomal dominant manner. However, most acquire the disease via a spontaneous mutation *in utero* (1). Patients often present in infancy with developmental delay, acquired microcephaly, intractable seizures, and dystonia. Later in life, they may have ataxia, paroxysmal neurologic events, and paroxysmal exertion-induced dyskinesia with or without epilepsy (1). The hallmark of GLUT1 deficiency is low CSF glucose concentration with normoglycemia (2).

Wilson disease (WD) is an autosomal recessive disorder of copper metabolism caused by a mutation in the *ATP7B* (ATPase Copper Transporting Beta) gene on chromosome 13q14.3 (3, 4). Mutations in the *ATP7B* gene can lead to detrimental copper accumulation. The prevalence of specific mutations in Wilson disease varies by geographic location. The p.H1069Q mutation is one of the most common mutations and has a population allelic frequency of 10–40%. Other common mutations that exist in the *ATP7B* gene include p. E1064A, p.R778L, p.G943S, and p.M769V (5). Patients with WD typically present with liver disorder (40–60%) in children >2 years of age, neurological manifestations (40–50%) associated to Kayser-Fleischer (KF) rings (90–100%) in children >10 years of age, and psychiatric symptoms (10–25%) in children >15 years of age (6, 7). Early diagnosis and treatment of both GLUT1 deficiency and WD is crucial and drastically improves long-term outcomes. Here we describe a patient who presented with characteristics of both disorders.

## Case Description

We present a case with global developmental delay and ataxia due to GLUT1 that subsequently was diagnosed with WD due to persistently elevated liver enzymes. This patient was born from a non-consanguineous family. The mother was a 35-year-old healthy woman of Salvadoran descent, and the father, 32 years old, was of Cuban descent. While the father was diagnosed with type 1 diabetes mellitus at age 4, there was no family history of seizures, intellectual disability, hearing loss, pregnancy losses, neurocutaneous syndromes, or metabolic diseases.

The patient's developmental delay became evident at 18 months. His vocabulary was limited, and he did not walk until 20 months. Initial workup performed by the neurology service, including complete blood count (CBC), comprehensive metabolic panel (CMP), creatine phosphokinase test (CPK), and Fragile X, were all found to be normal. Magnetic resonance imaging (MRI) of the brain was unremarkable. At age 3, he started having ataxic episodes. Videographic assessment demonstrated episodes of falling to the floor due to a collapse of the right leg. He would try to stand but immediately fall back to the ground without loss of consciousness. Such ataxic episodes subsided but recurred at 5 years of age.

Video electroencephalography (vEEG) showed paroxysmal events of sudden abnormal movements with bilateral hand dyskinesia that were non-rhythmic and non-synchronous. However, the videos showed no electroencephalographic seizures or postictal state. Hence, the patient was diagnosed with paroxysmal exercise-induced dyskinesia.

Genetic testing revealed a pathogenic heterozygous mutation c.988 C>T (p.Arg330) in the exon 8 of the *SLC2A1* gene by ACMG criteria PVS1, PM2, and PP5. This variant creates a premature translational stop signal resulting in an absent or disrupted protein product. This loss of function in the *SLC2A1* gene has been previously reported in individuals with GLUT1 deficiency (2). Parental testing was negative for the *SLC2A1* gene mutation.

During this workup, increasing levels of aspartate aminotransferase (AST) and alanine aminotransferase (ALT) were discovered. A ketogenic diet for his GLUT1 deficiency could not be started because of unexplained elevated liver enzymes. Thus, he was placed on a low carbohydrate diet. An ultrasound of the abdomen showed a heterogeneous liver appearance and a contracted gallbladder. Laboratory results, including prothrombin time (PT), partial thromboplastin time (PTT), antithrombin III activity, protein C, protein S, lupus anticoagulation, cardiolipin antibody, homocysteine level, hepatitis panel, Epstein Barr virus serology, thyroid-stimulating hormone (TSH), lead level, tissue transglutaminase, and alpha 1 antitrypsin phenotype were all unremarkable. The patient was not on any hepatotoxic medication. Ceruloplasmin level was low at 15 mg/dL (NL 28.6–56.1 mg/dL for age and sex) (8) and a basal 24-h urinary copper excretion was elevated at 127 mcg/24 h (NL 15–60 mcg/24 h).

**Figure 1:**
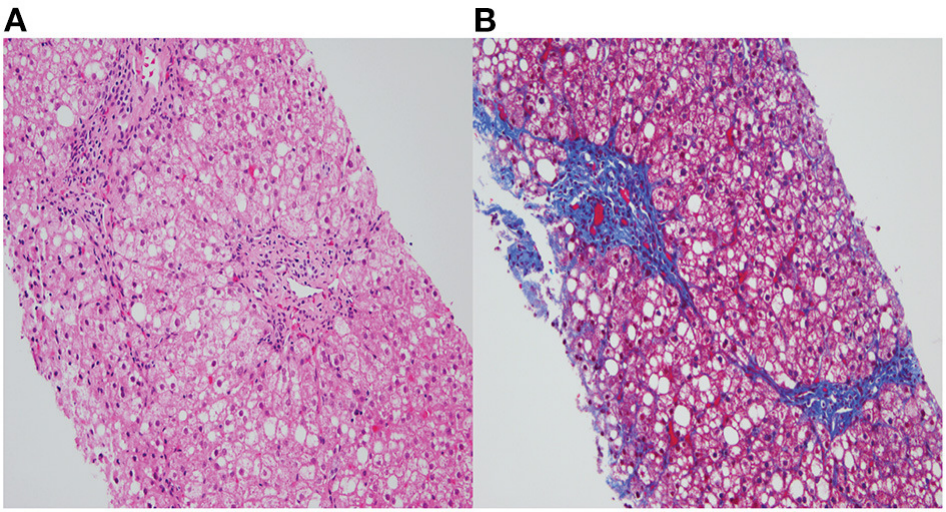
**(A)** Fatty change and ballooned hepatocytes with mild portal chronic inflammation (H&E, X200). **(B)** Portal fibrosis with slender septa extending from portal tracts and surrounding steatosis (Trichrome stain, X200).

Ophthalmologic exam was unremarkable. Repeat MRI of the brain was normal. The liver biopsy showed moderate (50–60%) macrovesicular steatosis, mild portal inflammation with lymphocytes and eosinophils, and grade II to III periportal fibrosis with slender portal septa (Figures 1A,B). The hepatic parenchymal copper concentration was 1,243 mcg/g dry weight (NL 10-35 mcg/g), suggesting WD. The diagnosis was confirmed by genetic testing, which revealed two pathogenic heterozygous variants of the *ATP7B* gene. One *ATP7B* allele showed a c.3207C>A (p.H1069Q) transversion on exon 14, which causes a substitution of histidine (CAC) to glutamine (CAA), and classified as pathogenic by ACMG criteria PM1, PP3, and PP5 (4). The second *ATP7B* allele showed c.3263 T>A (p.Leu1088Ter) on exon 15, which creates a pre-mature stop codon causing premature protein truncation, and classified as pathogenic by ACMG criteria PVS1, PP5, PM2, and PP3. Both variants have been reported to be associated with WD (9). Additionally, both parents demonstrated to be carriers for WD upon parental testing.

Management of WD was initiated at the time of diagnosis. The patient was started on a copper-restricted diet, trientine hydrochloride 250 mg BID, and zinc acetate 25 mg BID, eventually leading to decreased urine copper excretion and normalization of liver enzymes.

**Figure 2:**
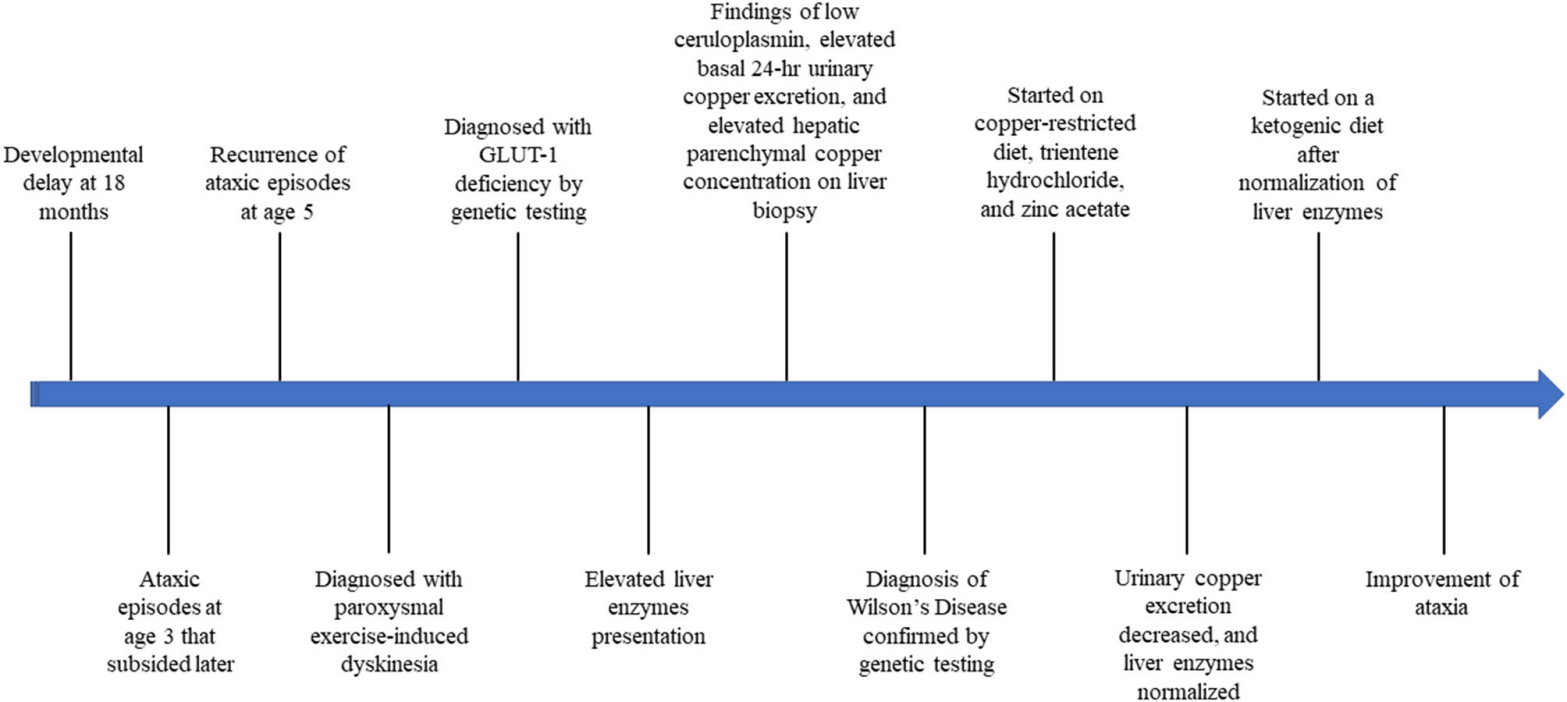
Timeline of our patient's presentation throughout diagnosis and treatment of Wilson disease and GLUT-1 deficiency.

Management of the GLUT1 deficiency with a ketogenic diet was initiated after normalization of his liver enzymes, which resulted in improvement of ataxia and seizures (Figure 2).

## Discussion

After an extensive literature review, we were unable to identify any other reported cases with an association with both WD and GLUT1 deficiency. These two disorders have different genetic modes of inheritance and two different loci on two different chromosomes. Simultaneous occurrence of an autosomal dominant disorder with an autosomal recessive disorder is extremely rare, especially with no history of consanguinity in the family. The presence of GLUT1 deficiency in our patient is most likely due to a *de novo* mutation, as a great number of *de novo* mutations occur in dominant genetic disorders (10). Moreover, the presence of WD in our patient could be related to parents coming from different countries of origin but Hispanic in nature.

Based on this patient's age of presentation and hepatic and neurological involvement, metabolic liver disorders including mitochondrial depletion syndrome (MDS) and Niemann-Pick disease type C (NPC) must be included in the differential diagnosis. MDS is associated to a group of autosomal recessive disorders characterized by a reduction in mitochondrial DNA, thus limiting energy production in several organs including the musculoskeletal, liver, and brain. Presentation includes seizures, hypotonia, developmental delay, feeding problems, and liver dysfunction (11). Moreover, NPC is a lysosomal disorder whose presentation is age-dependent, progressing from early perinatal and infantile liver involvement to hypotonia, developmental delay, ataxia, seizures, and psychiatric manifestations (12). Both disorders are diagnosed by molecular genetic testing.

This patient's early developmental delay presentation led to an extensive neurological workup and diagnosis of paroxysmal exertional dyskinesia with a seizure component secondary to GLUT1 deficiency. Cerebellar ataxia might present later in life in almost 30% of WD patients. These patients can present with an ataxic gait (wide-based gait with tandem walking), intentional tremor, dysdiadochokinesis, impaired coordination of fine hand movements, and ataxic speech (13–15). Moreover, KF rings, a copper deposition at the cornea's Descemet membrane, appear in 90–100% of patients with WD and neurological and psychiatric symptoms in >10 years of age (6). However, such type of ataxia and ocular findings were not seen in our patient.

Although pediatric WD commonly presents in the first decade of life, WD's neurological symptoms are mostly observed in the second decade (16, 17). Therefore, his neurological symptoms were most likely secondary to GLUT1 deficiency. GLUT 1 serves as a transporter in the central nervous system (CNS), and, in its absence, the patient will present with a normal blood glucose concentration and a CSF glucose concentration of <60 mg/dL (1). The prognosis of GLUT1 deficiency varies, but most respond well to a ketogenic diet. A ketogenic diet comprises a high-fat and low-carbohydrate diet, which maintains ketosis and allows the brain to use the ketone bodies as an alternative energy source. Ketogenic diets are currently considered the sole treatment for those with GLUT1 deficiency (2, 18). Liver enzymes alongside abdominal ultrasonography need to be closely monitored as studies have shown that a long-term ketogenic diet can induce parenchymal liver injury, hepatic steatosis, and formation of gallstones (19).

Moreover, our patient had elevated liver enzymes prior to initiation of ketogenic diet, likely related to WD and not usually consistent with GLUT1 deficiency. WD is caused by a mutation in the *ATP7B* gene, which encodes a copper transporting P-type ATPase necessary for copper excretion through the plasma and the bile. ATP7B is highly expressed in the liver but is also found in other organs such as the kidney, placenta, mammary glands, brain, and lung (5). If such a defect exists in the *ATP7B* gene, it can lead to a progressive toxic copper accumulation in these organs. Most children with WD present with liver disease manifesting with incidental and asymptomatic elevated liver enzymes, hepatomegaly, acute hepatitis, or cirrhosis. If WD is not appropriately diagnosed and treated, such copper accumulation can lead to liver failure and/or irreversible brain damage (7). Therefore, WD must be considered in the differential diagnosis of a patient with unexplained elevated liver enzymes, in the presence of another confirmed genetic condition since delay in diagnosis will delay early treatment. Recent studies have demonstrated the importance of performing genetic sequencing of the ATP7B gene in the initial investigation (20).

## Conclusion

This case highlights the importance of carefully evaluating a patient with an unexplained liver disorder, even in the presence of primary neurological disease, as it can have significant therapeutic implications.

## Data Availability Statement

The original contributions presented in the study are included in the article/supplementary material, further inquiries can be directed to the corresponding author/s.

## Ethics Statement

Written informed consent was obtained from the relevant individual(s), and/or minor(s)' legal guardian/next of kin, for the publication of any potentially identifiable images or data included in this article.

## Author Contributions

JD, AGF, and AF contributed with the elaboration, drafting, and final approval of the work. RA, MG, PJ, PS, and EH contributed with the revision and final approval of the work, and offered professional guidance. All authors agree to be accountable for all aspects of this work.

## Conflict of Interest

The authors declare that the research was conducted in the absence of any commercial or financial relationships that could be construed as a potential conflict of interest.

## Publisher's Note

All claims expressed in this article are solely those of the authors and do not necessarily represent those of their affiliated organizations, or those of the publisher, the editors and the reviewers. Any product that may be evaluated in this article, or claim that may be made by its manufacturer, is not guaranteed or endorsed by the publisher.

## Abbreviations

ALT: Alanine aminotransferase
ATOX1: Antioxidant protein 1
AST: Aspartate aminotransferase
*ATP7B*: ATPase Copper Transporting Beta
CTR1: Cell through copper transporter 1
CNS: Central Nervous System
CBC: Complete blood count
CMP: Comprehensive metabolic panel
CPK: Creatine phosphokinase test
GLUT1: Glucose transporter type 1
KF: Kayser-Fleischer
MRI: Magnetic resonance imaging
MDS: Mitochondrial depletion syndrome
NPC: Niemann-Pick disease type C
PTT: Partial thromboplastin time
PT: Prothrombin time
*SLC2A1*: Solute Carrier Family 2 Member 1
TSH: Thyroid-stimulating hormone
TGN: Trans-Golgi network
vEEG: Video electroencephalography
WD: Wilson disease.
